# Dr. Walker, on Vaccination

**Published:** 1808-05

**Authors:** John Walker


### *To the Editors of the Medical and Physical Journal.*

GENTLEMEN,

*Salisbury Court*, 8, *iv*, 1808.

IT may seem superfluous, at this time of day, to offer any thing more on the simple subject of Vaccination and its efficacy. Yet, as mistaken and mischievous men continue, in various ways, to excite alarms among the multitude; and as you may have readers sufficiently liberal to set some value on the testimony of an individual who has 'seen service,' more than most, in this easiest of all professional duties, I offer to you one of the many answers I have had to make to the solicitous enquiries continually addressed to me.

J. W.

'JOHN WALKER to GEORGE VANSITTART, very respectfully.

FLATTERED by thy call on me for a declaration on the efficacy of Vaccination in preventing the Small-Pox, for the satisfaction of a lady in the country, alarmed by the prejudices which have been excited through the evil reports which have been so mischievously propagated agianst it, it, I have great pleasure in presenting to thee a copy of the evidence given by me on the subject to the Royal College of Physicians.

"Q. 1. What have been your opportunities of acquiring knowledge respecting Vaccination, and what number have you vaccinated?

A. Since his return to England, at the close of 1801, has vaccinated upwards of 10,000, and has distributed upwards of 46,000 charges of matter to upwards of 11,000 applicants.

Q. 2. What success has attended the practice, and have any failures in its preventive power, or other bad effects followed?

A. His success has been quite equal to the report of the most strenuous advocates for Vaccination. No failure in its prevention of Small-pox has happened after he has declared the Vaccination to be perfect; and, in his own practice, he has never experienced any bad effects from it.

Q. 3. Is there any peculiarity in your practice of Vaccination?

A. He thinks there is. He is in the habit of puncturing the vesicle from the earliest period, without any diminution of effect, and he thinks (when the pock is near its height) with advantage to the patient.

He has occasionally taken effectual matter as late as the 15th day, and has declined the use of matter as early as the 8th day from a pock which has seemed too much advanced at that period.

He is cautious in taking his matter, to wipe away any pus which may have formed under the scab, which sometimes appears on the centre, and is distinct from the surrounding vaccine fluid.*

Q. 4. What cutaneous diseases have occurred as a consequence of Vaccination, and at what period?

---

* My reason for supposing there was any thing peculiar in my practice of vaccination, (for who would not as naturally wipe away pus from a vaccine pock in order to obtain limpid ichor as he would skim off with his hand the leaves, straw, or chaff on the surface of the water, at a spring where he wished to drink?) has arisen out of the letter of Dr. Jenner to the Medical Council of the Jennerian Society, wherein he says, "What will the public think on comparing the instructions given by Dr. Walker with those given by me?" &c. I mention this as an apology to your medical readers who may be ready to wonder how any inoculator can get it into his head that there can be any peculiarity in his practice of this most simple of all things appertaining to the healing art.——Medical Journal, Vol. XII, pp. 243, 303, 440.

A. Has

A. Has no recollection of having seen any consequent cutaneous affection, which could be attributed to Vaccination in his own practice in this country.

(Copy, signed R. Powell, January 22, 1807.)"

In addition to the evidence, which the lady must suppose to have not been rashly or unguardedly given, as being offered to the greatest authority, I will endeavour, by the mention of a fact of frequent occurrence, to shew that while the most obvious effect of the inoculation is visible only at the part where the lancet is applied, the whole skin is really acted upon; and, therefore, that the security ought to be, (which it really is,) as complete as if the subject had been covered with the Small-Pox from head to foot.

I usually inoculate my patients on each arm. It sometimes happens that the infection only takes in one arm. In a week, or less, I renew the operation on the arm where it has not taken. On this arm the process is quicker than it was on the other. It nearly overtakes the other in its progress; at the same time, it does not quite equal the former inoculation in its effect.—Thus, the skin is not found in the same condition at the time of the latter inoculation as it was at the time of the former; *ergo*, 'the whole skin is really acted upon, and, therefore, the security ought to be (as it really is) as complete as if the subject had been covered with the Small-Pox from head to foot.' Farewell.

*London, 22nd of Third Month, (March,)* 1808.

(Geo. Vansittart, Esq. M. P.)

